# High-dose folic acid supplementation results in significant accumulation of unmetabolized homocysteine, leading to severe oxidative stress in *Caenorhabditis elegans*

**DOI:** 10.1016/j.redox.2020.101724

**Published:** 2020-09-15

**Authors:** Kyohei Koseki, Yukina Maekawa, Tomohiro Bito, Yukinori Yabuta, Fumio Watanabe

**Affiliations:** a The United Graduate School of Agricultural Sciences, Tottori University, 4-101 Koyama-Minami, Tottori City, Tottori, 680-8553, Japan; b Graduate School of Sustainability Science, Tottori University, 4-101 Koyama-Minami, Tottori City, Tottori, 680-8553, Japan

**Keywords:** *Caenorhabditis elegans*, Folate, Folic acid, Homocysteine, Oxidative stress

## Abstract

Using *Caenorhabditis elegans* as a model animal, we evaluated the effects of chronical supplementation with high-dose folic acid on physiological events such as life cycle and egg-laying capacity and folate metabolism. Supplementation of high-dose folic acid significantly reduced egg-laying capacity. The treated worms contained a substantial amount of unmetabolized folic acid and exhibited a significant downregulation of the mRNAs of cobalamin-dependent methionine synthase reductase and 5,10-methylenetetrahydrofolate reductase. In vitro experiments showed that folic acid significantly inhibited the activity of cobalamin-dependent methionine synthase involved in the metabolism of both folate and methionine. In turn, these metabolic disorders induced the accumulation of unmetabolized homocysteine, leading to severe oxidative stress in worms. These results were similar to the phenomena observed in mammals during folate deficiency.

## Highlights

• High-dose folic acid supplementation reduced egg-laying ability in worms.
• Substantial amounts of folic acid and homocysteine were accumulated in the worms.
• The mRNA expression of methylenetetrahydrofolate reductase was reduced in the treated worms.
• Folic acid was a potent inhibitor of cobalamin-dependent methionine synthase in in vitro tests.
• High-dose folic acid supplementation in worms resulted in severe oxidative stress.

## Graphical abstract

**Figure:**
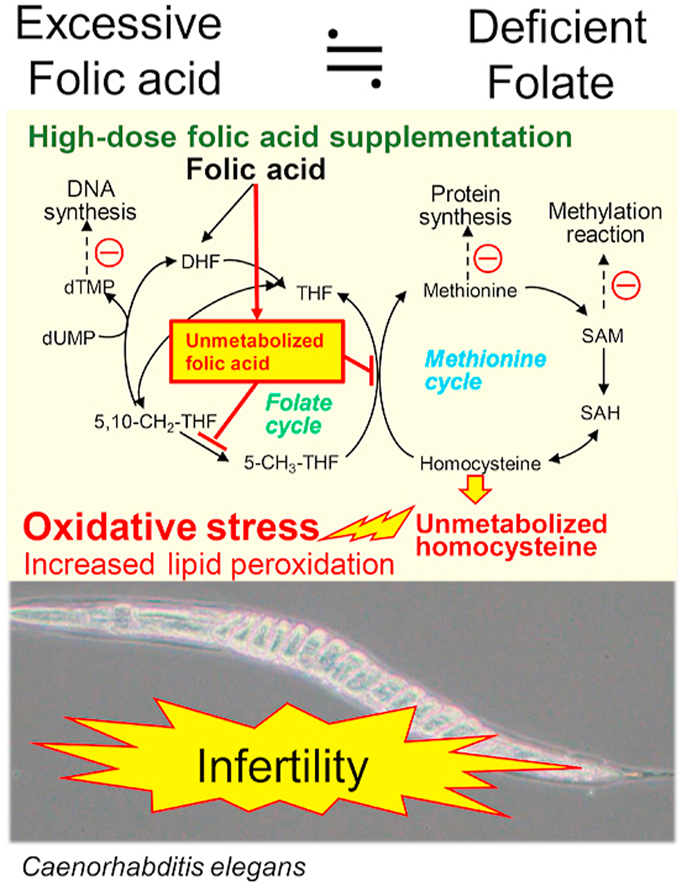

## 1 Introduction

**Fig. 1:**
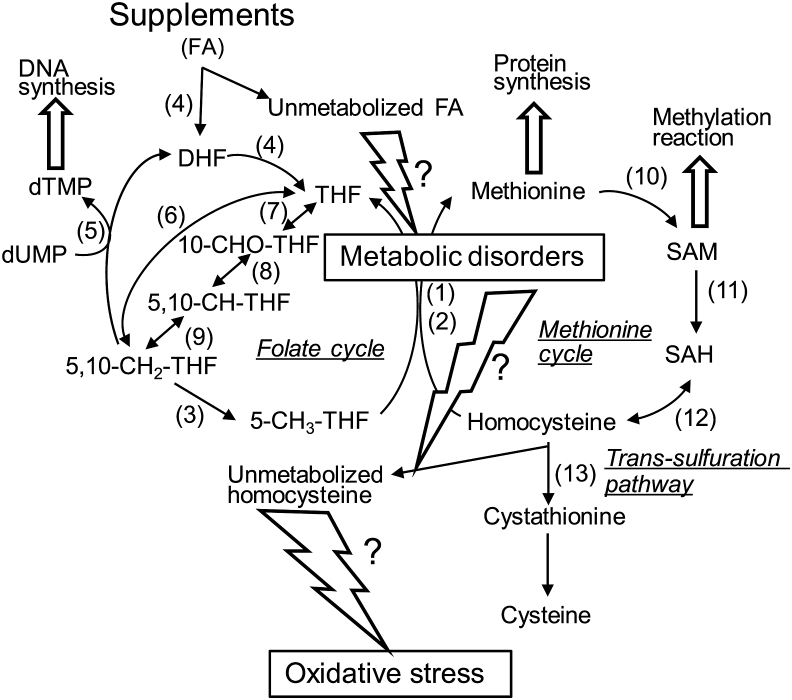
**Folate and methionine cycles in mammals and *C. elegans*.** FA, folic acid; DHF, dihydrofolate; THF, tetrahydrofolate; 5CH_3_-THF, 5-mentyltetrahydrofolate; 5,10-CH_2_-THF, 5,10-methylenetetrahydrofolate; 5,10-CH-THF, 5,10-methenyltetrahydrofolate; 10-CHO-THF, 10-folmyltetrahydrofolate; SAM, S-adenosylmethionine; SAH, S-adenosylhomocysteine; 1, cobalamin-dependent methionine synthase (*metr-1*); 2, methionine synthase reductase (*mtrr-1*); 3, methylenetetrahydrofolate reductase (*mthf-1*); 4, dihydrofolate reductase (*dhfr-1*); 5, thymidylate synthase (*tyms-1*); 6, serine hydroxymethyl transferase (*mel-32a*); 7, methenyltetrahydrofolate synthetase (K07E3.4); 8, mehenyltetrahydrofolate cyclohydrolase (*dao-3*); 9, mehylenetetrahydrofolate dehydrogenase (K07E3.4); 10, methionine adenosyltransferase (*sams-1*); 11, methyltransferases (not identified); 12, adenosine homocysteinase (*ahcy-1*); 13, cystathionine β-synthase (*cbs-1*). The parentheses represent the orthologous genes encoding human folate and methionine metabolic enzymes in *C. elegans*.

Folate refers to a group of water-soluble vitamins that are essential for human health and development [1]. Folate compounds participate in one-carbon metabolism, which serves to activate and transfer one-carbon units for biosynthetic reactions, including purine and thymidine synthesis and homocysteine remethylation [1]. Folate deficiency leads to reproductive impairments, including impaired fetal development [2]. Because of this impaired folate status, the prevalence of neural tube defects in newborn increased [2]. Supplementation with folic acid, a synthetic and stable form of folate, during the periconceptional period is effective in reducing neural tube defects [3]. Moreover, folic acid supplementation reportedly has health-promoting effects as it prevents various diseases, such as type 2 diabetes and cardiovascular diseases (including stroke) [4]. However, excessive folic acid intake has been linked to the masking of cobalamin deficiency [5], and concern is now growing with regarding the adverse effects of long-term intake of high-dose synthetic folic acid [[6], [7], [8], [9]]. In fact, high-dose folic acid supplementation has resulted in an increase in folic acid levels in the blood circulation [10,11]. Because folic acid is a synthetic compound that has no biological function unless it is reduced to dihydrofolate and tetrahydrofolate [12,13], the folic acid that accumulates in this way has been termed unmetabolized folic acid. If the concentration of unmetabolized folic acid reaches a high level in cells, it might induce metabolic disorders of the folate and methionine metabolic cycles, leading to the formation of unmetabolized homocysteine, which acts as a pro-oxidant (Fig. 1). However, there is a lack of information on the biochemical and physiological consequences of the excessive unmetabolized folic acid formed by chronic supplementation with folic acid, and on whether chronical supplementation with high-dose folic acid is beneficial or harmful.

*Caenorhabditis elegans* has a very short life cycle and has the ability to change its locomotor behavior, reproductive rate, and lifespan. Worms conserve various of the molecular processes and cellular metabolisms of mammals [14]. In fact, *C. elegans* has a series of orthologous genes that encode enzymes involved in the mammalian folate and methionine cycles, as shown in Fig. 1 [[15], [16], [17], [18], [19], [20], [21], [22]]. Thus, worms would be used as a model animal to reach an improved understanding of the relationship between biological events and supplementation with folic acid.

Here, we described the effects of chronic high-dose folic acid supplementation on egg-laying capacity in worms and elucidated the manner in which the formed excessive unmetabolized folic acid disrupted folate metabolism to form unmetabolized homocysteine as a potent pro-oxidant.

## 2 Methods

### 2.1 Organisms

The N2 Bristol wild-type *C. elegans* strain was grown at 20 °C on Petri dishes containing nematode growth medium (NGM). The *Escherichia coli* OP50 strain was used as food [23]. One egg from worms grown on NGM plates in the presence of OP50 *E. coli* was transferred onto a plate containing NGM supplemented with folic acid (purity >97%; 0, 0.3, 2.9, and 8.8 μmol/plate; Sigma-Aldrich, St. Louis, MO, USA). The egg was allowed to hatch and develop into an egg-laying adult worm. After the adult worm was removed from each plate, each egg collected was transferred onto a new folic acid-supplemented plate. After repeating this procedure five times under the same conditions, the worms were used in the experiments to evaluate the chronic effects of folic acid supplementation on the worms. Worms that were grown in the absence of folic acid supplementation were used as controls. Folic acid-supplemented worms were transferred to NGM (without folic acid supplementation) for three generations and used as recovery worms.

### 2.2 Preparation of recombinant human folate conjugase

A recombinant human folate conjugase was prepared to examine the presence of folate compounds in worms. The detailed procedures used for the preparation of this enzyme are described in the **Supplemental Material**.

### 2.3 Determination of total folates using a microbiological method

Worms grown under folic acid-supplemented or control conditions were incubated for 1 h at 20 °C in fresh NGM, to remove *E. coli* cells. Worms (2 g wet weight) were homogenized using a hand homogenizer and sonicator. The homogenate was suspended in 1.5 mL of 0.1 mol/L potassium phosphate butter, pH 6.1, and boiled for 10 min. The extract was treated with protease (actinase E, Nacalai Tesque, Kyoto, Japan) and folate conjugase, followed by centrifugation at 15,000×*g* for 15 min at 4 °C. The supernatant fraction was used to determine the total folate content via a *Lactobacillus rhamunosus* ATCC 27773 (American Type Culture Collection, Manassas, VA, USA) bioassay, according to the Japanese Standard Tables of Food Composition [24].

### 2.4 Determination of folic acid, tetrahydrofolate, and 5-CH_3_-tetrahydrofolate by high performance liquid chromatography (HPLC)

Folate compounds were extracted from worms according to the method of Nakata [25]. Control and high-dose folic acid-supplemented worms (0.05 g wet weight) were homogenized in 100 μL of 100 mmol/L potassium phosphate buffer, pH 6.1, containing 1% (w/v) ascorbic acid and 0.1% (v/v) mercaptoethanol on ice using a hand homogenizer. The homogenates were boiled for 10 min and then placed on ice. Folate conjugase (5 μL) was added to the treated homogenates, followed by incubation at 37 °C for 5 h under a nitrogen atmosphere. The treated homogenates were centrifuged at 15,000×*g* for 10 min at 4 °C. The supernatants were filtered through a membrane filter (Millex®-LH, 0.45 μm, Merck-Millipore, Burlington, MA, USA) used for HPLC samples. The concentrations of folic acid, tetrahydrofolate, and 5-CH_3_-tetrahydrofolate in worms treated with or without high-dose folic acid supplementation were determined using a Shimadzu (Kyoto, Japan) HPLC apparatus (SCL-10A VP system controller, DGU-20A3R degassing unit, LC-20AB liquid chromatograph, and CTO20AC column oven), according to the method of Patring et al. [26]. The separation of folate compounds was performed on μBondasphere C4 5 μm, 100A (150 × 3.9 mm I. D.; Waters, Milford, MA, USA) at 23 °C. The flow rate was 0.4 mL/min. For the detection and quantification of tetrahydrofolate and 5-CH_3_-tetrahydrofolate (Schircks Laboratories, Jona, Switzerland), a fluorescence detector (RF-530, Shimadzu) was used (excitation at 290 nm and emission at 360 nm), whereas, a UV/Vis detector (SPD-10AV, Shimadzu) set at 290 nm was used for the detection and quantification of folic acid. The mobile phase used here was acetonitrile/30 mmol/L phosphate buffer, pH 2.3, under linear-gradient elution conditions, as described in the cited references. The retention times of authentic tetrahydrofolate, 5-CH_3_-tetrahydrofolate, and folic acid were 12.5, 15.5, and 24.5 min, respectively.

### 2.5 Cell homogenate preparation

Control and folic acid-supplemented worms (0.05 g wet weight) were disrupted in 500 μL of 100 mmol/L potassium phosphate buffer, pH 7.0, on ice using a homogenizer. The homogenates were centrifuged at 15,000×*g* for 10 min at 4 °C. The supernatant fractions were used as crude enzymes or crude homogenates.

### 2.6 Oxidative stress marker assays

Hydrogen peroxide (H_2_O_2_) and malondialdehyde (MDA) concentrations were determined using an H_2_O_2_ assay kit (BioVision, Inc., Milpitas, CA, USA) and a TBARS assay kit (ZeptoMetrix Crop., Buffalo, NY, USA), respectively. These reaction products were assayed by measuring absorbance at 570 nm and 540 nm, respectively, using a microplate reader (Tecan Group Ltd., Männedorf, Switzerland). These markers were determined according to the respective manufacturer’s instructions.

### 2.7 Other assays

SAM and *S*-adenosylhomocysteine (SAH) were assayed by HPLC as described previously [27]. The activities of cobalamin-dependent methionine synthase [28] and 5,10-methylenetetrahydrofolate reductase [29] were assayed using the HPLC methods cited in the references. In the assay of cobalamin-dependent methionine synthase activity, the tetrahydrofolate formed by the enzymatic reaction of crude enzyme and 5-CH_3_-tetrahydrofolate, as a substrate (0.25 mmol/L), was assessed by measuring the fluorescence intensity at an excitation wavelength of 290 nm and an emission wavelength of 360 nm. In the assay of 5,10-methylenetetrahydrofolate reductase activity, the 5-CH_3_-tetrahydrofolate formed by the enzymatic reaction of crude enzyme and 5,10-methylenetetrahydrofolate, as a substrate (1 mmol/L), was assessed by measuring the fluorescence intensity at an excitation wavelength of 290 nm and an emission wavelength of 360 nm.

### 2.8 H_2_O_2_ fluorescent staining

To visualize H_2_O_2_ in control and folic acid-supplemented (8.8 μmol/plate) worm bodies, BES-H_2_O_2_-Ac (Wako pure chemical industry Corp., Ltd., Tokyo, Japan) was used as a fluorescent probe. The probe was dissolved in sterilized NGM at a final concentration of 200 μmol/L and used as a staining solution. Worms (approximately 300 worms each) grown under control and folic acid-supplemented conditions were treated with 150 μL of the staining solution for 1 h under aseptic conditions. Each worm was transferred onto fresh NGM and incubated at 20 °C for 1 h, to remove the staining solution from the surface of worm body. The washed worms were treated with 20 μL of 1 mmol/L sodium azide solution on a slide glass and observed using a fluorescence microscope (λ_ex_ = 485 nm, λ_em_ = 530 nm).

### 2.9 Quantitative polymerase chain reaction (qPCR) analysis

Total RNA was extracted from worms using Sephasol®-RNA1 (Nacalai Tesque). Poly(A)^+^ mRNA was purified from total RNA using the Poly (A)^+^ Isolation Kit from Total RNA (Nippon Gene, Tokyo, Japan), and was then used to synthesize cDNA using a PrimeScript™ II 1st Strand cDNA Synthesis Kit (Takara Bio, Otsu, Japan). To obtain 20–22 nucleotides in length with amplified products of approximately 100 bp, primer pairs were prepared using the GENETYX software (GENETYX Corp., Tokyo, Japan) (Table 1). qPCR was performed using a CFX Connect™ Real-Time System (Bio-Rad) with SYBR Premix Ex Taq (Takara Bio). The level of the mRNA encoding β-actin was used as an internal standard. The qPCR experiments were repeated at least three times for each cDNA obtained from three preparations of worms.

**Table 1:** **Primer pairs used for the qPCR analysis**. The primer pairs that were used for the qPCR analysis were designed using the GENETYX software. A full complement of homologs of the mammalian antioxidant enzymes has been identified in the genome of *C*. *elegans*, including cobalamin-dependent methionine synthase (*metr-1*), methionine synthase reductase (*mtrr-1*), methylenetetrahydrofolate reductase (*mthf-1*), dihydrofolate reductase (*dhfr*-*1*), thymidylate synthase (*tyms-1*) and serine hydroxymethyl transferase (*mel*-*32a*). The β-actin (*act-1*) mRNA levels served as the internal standard.

Gene name	Primer sequences (5′–3′)
*metr-1*	CCATTGGACCTACGAACAGAAC and ACAGTTTCCACGAGAAGCAC
*mtrr-1*	AAGACCGCTGATTCGTGTACTC and GCATGTCAGCCAATGACAATCC
*mthf-1*	TCGATTCTCTGTCCACAATGCC and TGCTTTGGTGACCAGCTCTT
*dhfr-1*	GCAGTATTTCGCGTCTGTTACC and CCAGCATTTTCGTCCGATGA
*tyms-1*	GGAACACGAAGAGATGATCGCA and ATTCCTCGAGAACTCCCTTCCA
*mel*-*32a*	AAGTTCAGCGCCACAAGTACAC and GTGAAGTTCTCGGAAGCGATGAG
*act-1*	TCCAAGAGAGGTATCCTTACCC and CTCCATATCATCCCAGTTGGTG

### 2.10 Analyses of egg-laying capacity and life cycle

Egg-laying capacity was measured according to the method of Byerly et al. [30]. L4-stage worms grown under folic acid-supplemented or control conditions were selected, transferred onto the respective new medium, and incubated for 24 h at 20 °C. After each worm was removed from the plate, the eggs laid were counted in triplicate.

The life cycle of control and folic acid-supplemented worms were assayed at 20 °C according to the method of Johnson and Wood [31].

### 2.11 Protein assay

Proteins were determined using ovalbumin as a standard, according to the method of Bradford [32].

### 2.12 Statistical analysis

All data, with the exception of the *C. elegans* H_2_O_2_-staining experiments, were evaluated by one-way ANOVA, and a post-hoc analysis was performed using Tukey’s multiple comparison tests on GraphPad Prism 3 for Windows version 2.01 (GraphPad software Inc., La Jolla, CA, USA), and were presented as the mean ± SEM. Differences were considered statistically significant at *P* < 0.05.

## 3 Results

### 3.1 Effect of folic acid supplementation on folate content in C. elegans

**Fig. 2:**
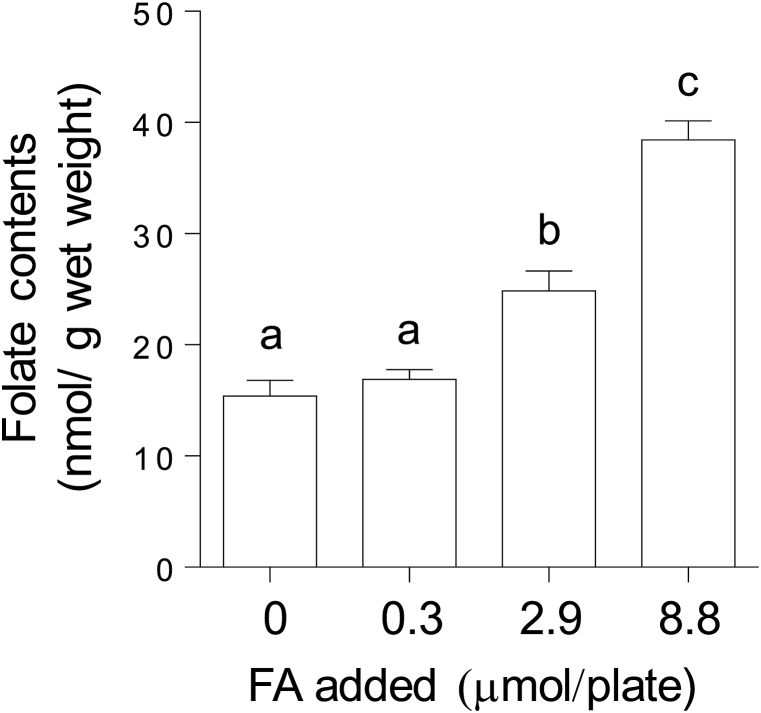
**Effect of various levels of folic acid supplementation of NGM on folate content in *C. elegans*.** Plates containing the indicated amounts of folic acid (FA)-supplemented NGM each received one egg, which was obtained from worms grown on NGM plates in the presence of OP50 *E. coli*. Eggs were allowed to hatch and develop into egg-laying adult worms. The adult worms were then removed from each plate, eggs were collected, and each egg was transferred onto a new folic acid-supplemented plate. After repeating this procedure five times under the same conditions, the worms were used to determine total folate using a *L. rhamunosus* ATCC 27773 microbiological method. Data represent the mean ± SEM of four independent experiments. Different letters (a–c) indicate values that are significantly different (*P* < 0.05); identical letters indicate values that are not significantly different.

To investigate the effects of chronic folic acid supplementation on various physiological functions in *C. elegans*, worms were grown for five generations under various folic acid-supplemented conditions (0, 0.3, 2.9, and 8.8 μmol/plate). The ingredients of NGM and *E. coli* as the diet for worms did not affect the concentrations of the supplemented folic acid because their folate levels were significantly lower than the supplemented folic acid levels (data not shown). Although 0.3 μmol/plate of folic acid supplementation did not affect the folate level of worms, 2.9 and 8.8 μmol/plate of folic acid supplementation significantly increased folate levels of worms (Fig. 2); in particular, the body folate level was increased by more than 2-fold in the worms supplemented with 8.8 μmol/plate of folic acid vs. the control worms.

### 3.2 Effects of folic acid supplementations on life cycle and egg-laying capacity

**Fig. 3:**
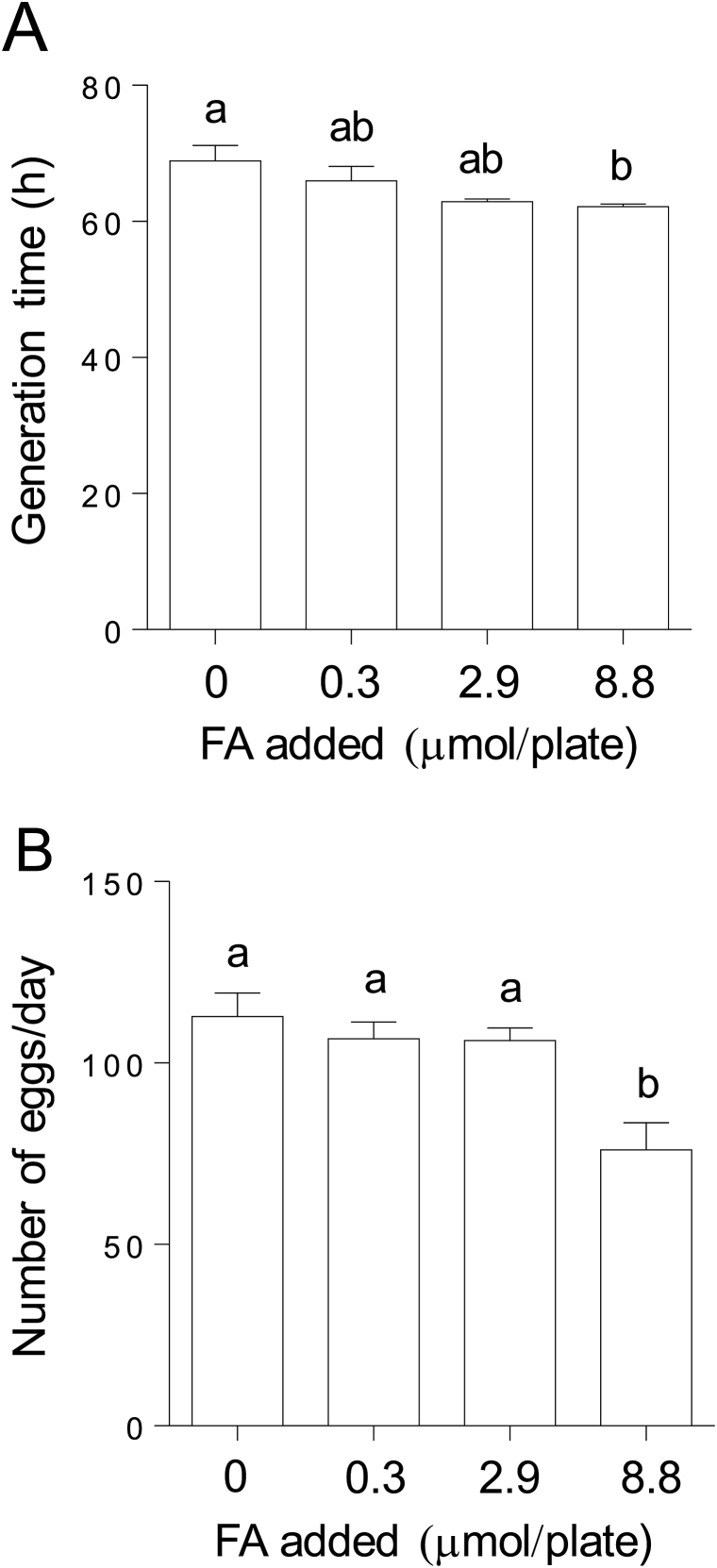
**Effects of folic acid-supplemented growth conditions on life cycle and egg-laying capacity in *C. elegans*.** (A) Length of the life cycle of *C. elegans* treated with the indicated amounts of folic acid. The total number of eggs laid per worm (B) was determined in worms grown in the presence or absence (control) of folic acid (FA) supplementation. Data represent the mean ± SEM of four independent experiments. The different letters (a, b) indicate values that are significantly different (*P* < 0.05); identical letters indicate values that are not significantly different.

High-level (8.8 μmol/plate) folic acid-supplemented worms had a shorter life cycle compared with the control and low (0.3 μmol/plate)- and moderate (2.9 μmol/plate)-level folic acid-supplemented worms (Fig. 3A). The total number of eggs laid was significantly decreased in high-dose folic acid-supplemented worms (Fig. 3B) whereas worms that received low and moderate folic acid supplementation did not exhibit changes in the total number of eggs laid. Thus, we used high-dose folic acid-supplemented (8.8 μmol/plate) worms in subsequent experiments.

### 3.3 Effects of high-dose folic acid supplementation on the levels of various cellular folate compounds

**Fig. 4:**
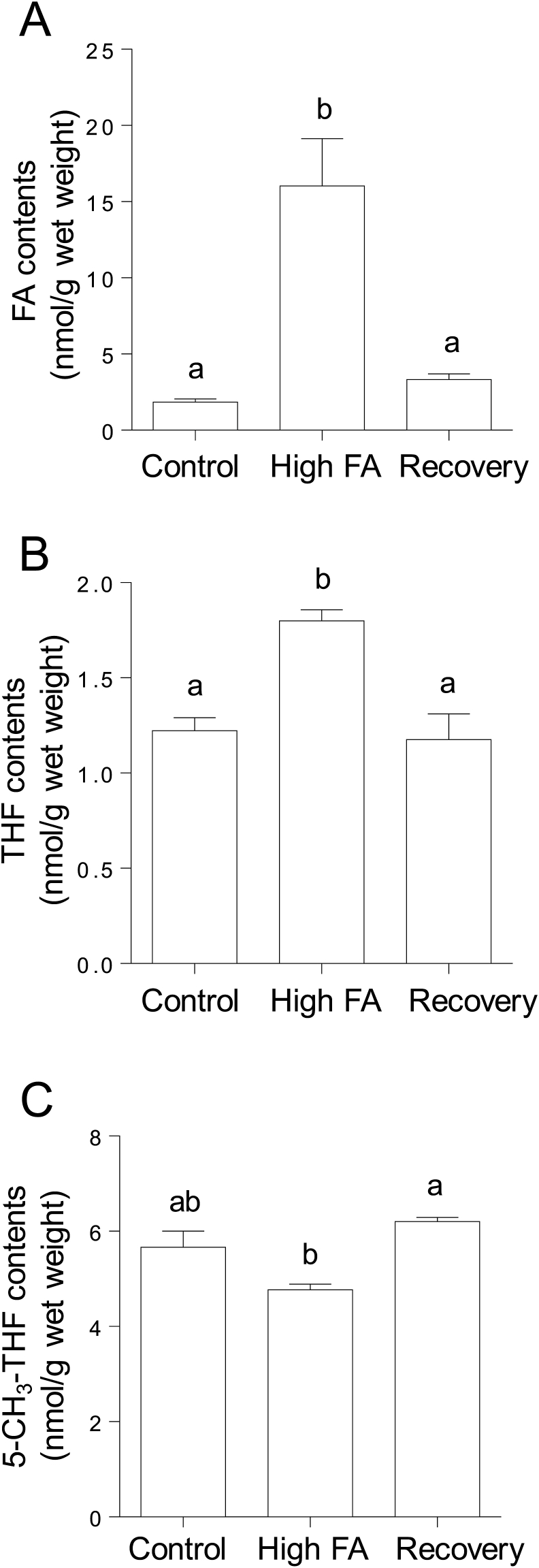
**Effects of folic acid supplementation on the levels of several cellular folate compounds in *C. elegans*.** Folic acid (or unmetabolized folic acid) (A), tetrahydrofolate (B), and 5-methyltetrahydrofolate (C) were determined in folic acid (FA)-supplemented (8.8 μmol/plate) and control worms using HPLC. After folic acid-supplemented worms were grown for three generations under control (without folic acid supplementation) conditions, these values were determined again in the worms (shown as “Recovery”). Data are presented as the mean ± SEM of three independent experiments. Different letters (a, b) indicate values that are significantly different (*P* < 0.05); identical letters indicate values that are not significantly different.

The folic acid content in worms grown under high-dose folic acid-supplemented conditions was determined. The folic acid content was increased by approximately 20-fold in high-dose folic acid-supplemented worms compared with the control worms (Fig. 4A). The significantly accumulated folic acid was recovered to the control level after the treated worms were grown for three generations under the control conditions. Although tetrahydrofolate was significantly increased by the supplementation with high folic acid, 5-CH_3_-tetrahydrofolate was decreased slightly after the same treatment. In worms, as well as in humans, 5-CH_3_-tetrahydrofolate is reportedly the predominant folate (approximately 60% of total folates) [33]. Using this percentage, the total folate content (approximately 8 nmol/g wet weight) of high-dose folic acid-supplemented worms was calculated from the values determined for 5-CH_3_-tetrahydrofolate. The ratio of folic acid/total folates was remarkably increased in the folic acid-supplemented worms (approximately 2) relative to the control (approximately 0.25). These results suggest that most of the folic acid taken up by worm cells is not metabolized to form dihydrofolate or tetrahydrofolate and remains as unmetabolized folic acid.

### 3.4 Effects of high-dose folic acid supplementation on the mRNA levels of folate metabolic enzymes

**Fig. 5:**
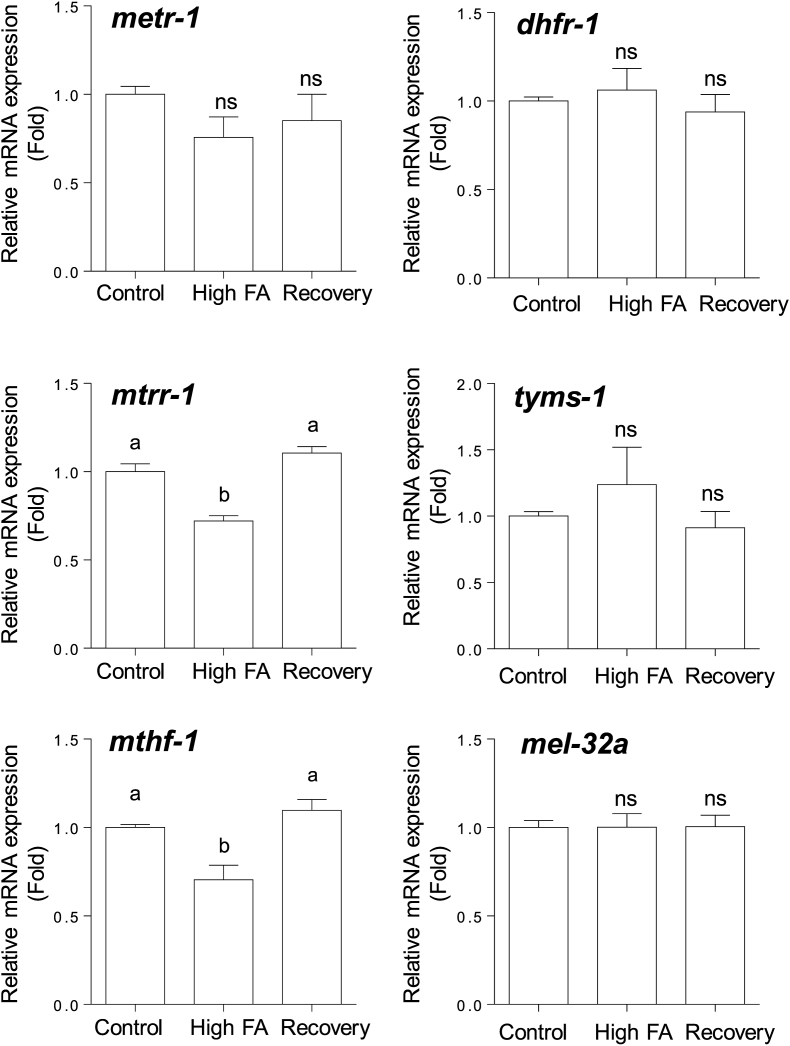
**Effects of folic acid supplementation on the levels of mRNAs encoding various enzymes involved in cellular folate metabolism in *C. elegans*.** Cobalamin-dependent methionine synthase (*metr-1*), methionine synthase reductase (*mtrr-1*), methylenetetrahydrofolate reductase (*mthf-1*), dihydrofolate reductase (*dhfr-1*), thymidylate synthase (*tyms-1*), and serine hydroxymethyl transferase (*mel-32a*) were determined in worms grown in the presence or absence (control) of folic acid (FA) supplementation (8.8 μmol/plate). After folic acid-supplemented worms were grown for three generations under control (without folic acid supplementation) conditions, these values were determined again in the worms (shown as “Recovery”). Data are presented as the mean ± SEM of three independent experiments. Different letters (a, b) indicate values that are significantly different (*P* < 0.05); identical letters indicate values that are not significantly different. ns, non-significant difference vs. the control.

To clarify the effect of high-dose folic acid supplementation on the folate metabolism of *C. elegans*, the expression levels of mRNAs for enzymes involved in the folate cycle were determined (Fig. 5). Remarkably, the expression levels of the cobalamin-dependent methionine synthase reductase and methylenetetrahydrofolate reductase mRNAs were decreased significantly in *C. elegans* during high-dose folic acid supplementation. The expression level of the cobalamin-dependent methionine synthase mRNA was decreased slightly. In turn, high-dose folic acid supplementation did not affect the levels of the dihydrofolate reductase, thymidylate synthesis, and serine hydroxymethyl transferase mRNAs. The changed mRNA levels were recovered to control levels after high-level folic acid-supplemented worms were grown for three generations under control conditions. These results indicated that high-dose folic acid supplementation significantly and specifically reduces the expression levels of the cobalamin-dependent methionine synthase reductase and methylenetetrahydrofolate reductase mRNAs.

### 3.5 Effects of various concentrations of folic acid on the activities of cobalamin-dependent methionine synthase and methylenetetrahydrofolate reductase

**Fig. 6:**
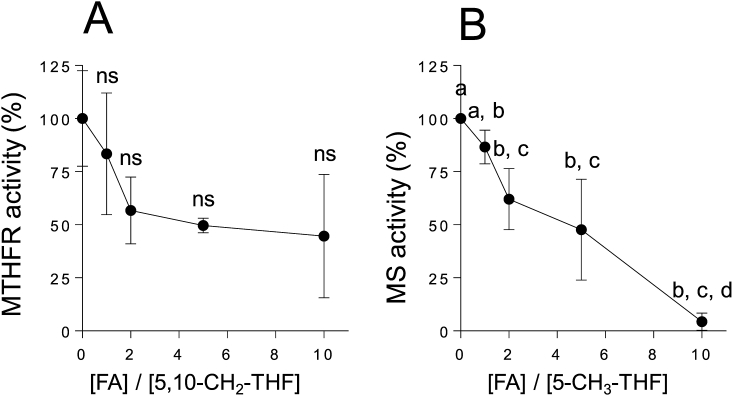
**Effects of various concentrations of added folic acid on the activities of methylenetetrahydrofolate reductase and cobalamin-dependent methionine synthase in a homogenate of worms.** A, methylenetetrahydrofolate reductase and B, cobalamin-dependent methionine synthase. The activities of the two enzymes are presented as relative activities (%) against the enzymatic activities recorded in the absence of folic acid. Substrate ratios, i.e., [FA]/[5,10-CH_2_-THF] and [FA]/[5-CH_3_-THF], in reaction mixtures represent [folic acid added]/[5,10-methylenetetrahydrofolate] and [folic acid added]/[5-methyltetrahydrofolate], respectively. Data are presented as the mean ± SEM of three independent experiments. Different letters (a–d) indicate values that are significantly different (*P* < 0.05); identical letters indicate values that are not significantly different. ns, non-significant difference vs. the control.

To examine whether unmetabolized folic acid has the ability to inhibit the activities of two key enzymes—cobalamin-dependent methionine synthase, which is involved in the methionine cycle, and methylenetetrahydrofolate reductase, which is involved in the folate cycle—we determined the effects of various concentrations of folic acid on their activities using a crude enzyme. In the presence of 10 mmol/L 5,10-CH_2_-tetrahydrofolate, as a substrate, the activity of methylenetetrahydrofolate reductase was decreased by approximately 45% of the [folic acid]/[5,10-CH_2_-tetrahydrofolate] concentration ratio of 2, and decreased slightly thereafter (Fig. 6A); however, this difference was not statistically significant.

Although the cobalamin-dependent methionine synthase activity was significantly decreased with increased concentrations of folic acid in the presence of 5 mmol/L 5-CH_3_-tetrahydrofolate, as a substrate, approximately 40% of its enzymatic activity was inhibited a [folic acid]/[5-CH_3_-tetrahydrofolate] concentration ratio of 2 (Fig. 6B). These results suggest that unmetabolized folic acid acts as a potent inhibitor of cobalamin-dependent methionine synthase, which is involved in the metabolism of folate and methionine.

### 3.6 Effects of high-dose folic acid supplementation on the levels of SAM as a modulator of the folate and methionine cycles in C. elegans

**Fig. 7:**
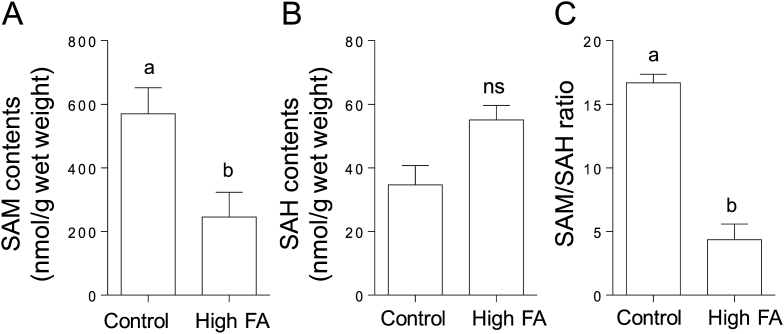
**Effects of high folic acid supplementation on the *S*-adenosylmethionine and *S*-adenosylhomocysteine content of *C. elegans.****S*-adenosylmethionine (SAM, A) and *S*-adenosylhomocysteine (SAH, B) were determined in high-dose folic acid (FA)-supplemented and control worms using HPLC, followed by the calculation of the SAM/SAH ratio (C). Data are presented as the mean ± SEM of three independent experiments. Different letters (a, b) indicate values that are significantly different (*P* < 0.05); identical letters indicate values that are not significantly different. ns, non-significant difference vs. the control.

To evaluate whether the reduced levels of expression of the cobalamin-dependent methionine synthase reductase and methylenetetrahydrofolate reductase mRNAs observed in high-dose folic acid-supplemented worms induce disorders of homocysteine metabolic pathways, SAM, SAH, and the SAM/SAH ratio were determined. High-dose folic acid supplementation significantly decreased SAM level and slightly increased SAH level in worms (Fig. 7A and B). The SAM/SAH ratio was significantly reduced in high-dose folic acid-supplemented worms compared with the control worms (Fig. 7C). These results suggest that high-dose folic acid supplementation induces the inhibition of the two homocysteine metabolic pathways, leading to the accumulation of unmetabolized homocysteine as a pro-oxidant.

### 3.7 Effects of high-dose folic acid supplementation on oxidative stress markers in C. elegans

**Fig. 8:**
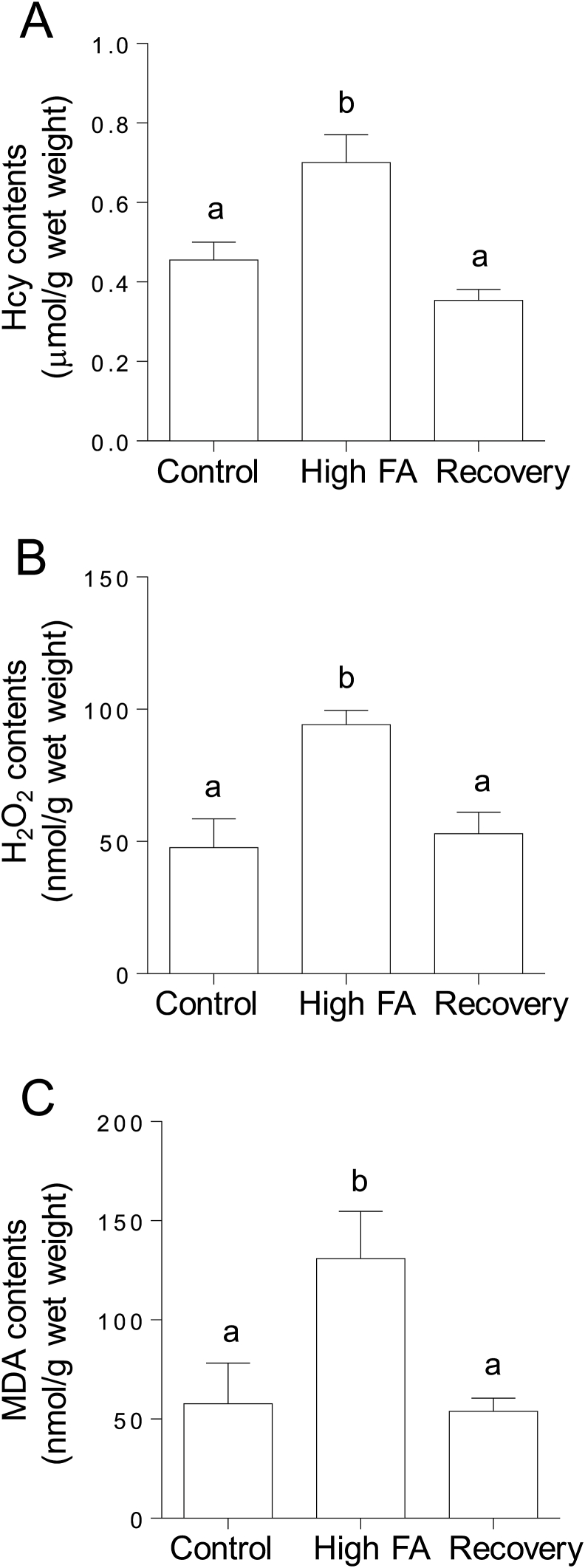
**Effects of folic acid supplementation on the levels of homocysteine and oxidative stress markers in *C. elegans*.** Homocysteine (A), hydrogen peroxide (H_2_O_2_, B), and malondialdehyde (MDA, C) were determined in folic acid (FA)-supplemented and control worms using HPLC and commercially available assay kits. After folic acid-supplemented worms were grown for three generations under control (without folic acid supplementation) conditions, these values were determined again in the worms (shown as “Recovery”). Data are presented as the mean ± SEM of three independent experiments. Different letters (a, b) indicate values that are significantly different (*P* < 0.05); identical letters indicate values that are not significantly different.

To clarify whether folic acid supplementation disrupts cellular redox homeostasis to induce oxidative stress, several biomarkers of oxidative stress were assayed in high-dose folic acid-supplemented worms. High-dose folic acid supplementation significantly increased homocysteine levels in worms (Fig. 8A). H_2_O_2_ and MDA (as a lipid peroxidation marker) levels were increased by approximately 2-fold in high-dose folic acid-supplemented worms compared with control worms (Fig. 8 B and C). The increased levels of homocysteine, H_2_O_2_, and MDA were restored to the control levels after folic acid-supplemented worms were grown for three generations under the control conditions.

**Fig. 9:**
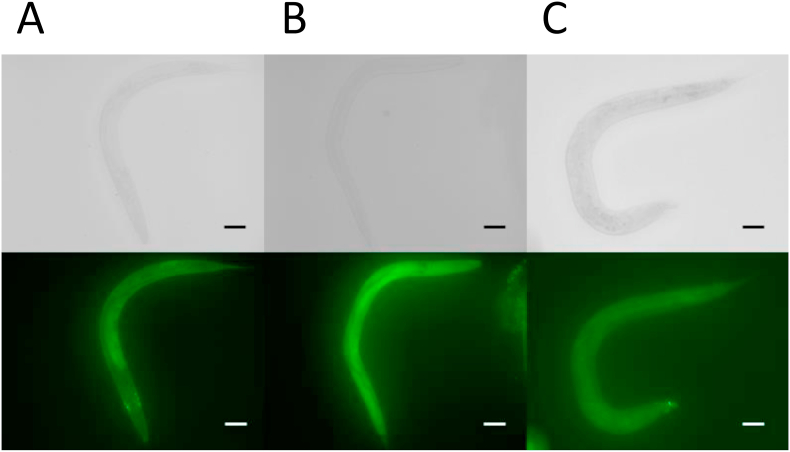
**Effect of folic acid supplementation on cellular H**_**2**_**O**_**2**_**in *C. elegans***. A fluorescent probe (BES-H_2_O_2_-Ac) was used to detect cellular H_2_O_2_ in control and folic acid (FA)-supplemented (8.8 μmol/plate) worms. Control and folic acid-supplemented worms (approximately 300 worms each) were treated with the fluorescent probe solution and washed as described in the text. The washed worms were treated with 20 μL of 1 mmol/L sodium azide solution on a glass slide and observed under a fluorescence microscope (λ_ex_ = 485 nm, λ_em_ = 530 nm). The upper and lower panels present original photos and fluorescent images, respectively (scale bar, 100 μm). After folic acid-supplemented worms were grown for three generations under control (without folic acid supplementation) conditions, these values were determined again in the worms (shown as “Recovery”). Data are presented as typical photographs of three independent experiments. A, control worm; B, folic acid-supplemented worm; C, worm recovered from folic acid supplementation.

When the control and folic acid-supplemented worms were treated with a fluorescent probe (BES-H_2_O_2_-Ac) for detection of H_2_O_2_, the intestinal tract and its neighboring regions were stained slightly in the control worms (Fig. 9), coinciding with the observation that considerable amounts of homocysteine, H_2_O_2_, and MDA were detected even in the control (Fig. 8). The intensity of the fluorescence was increased significantly in the high-level folic acid-supplemented worms. The increased fluorescent intensity was restored to the control level after high folic acid-supplemented worms were grown for three generations under control conditions. These results suggest that chronic high-dose folic acid supplementation in worms results in disordered redox homeostasis, leading to severe oxidative stress.

## 4 Discussion

Folic acid does not occur naturally in food because it is a synthetic compound. As folic acid is the most stable form of various folate compounds, it has been used in food fortification and dietary supplements for humans [34,35]. Folic acid has no biological function unless it is reduced to dihydrofolate and tetrahydrofolate [12,13] (Fig. 1). After folic acid (known as pteroylmonoglutamic acid) is absorbed in the intestine, it is rapidly converted to reduced folates, mainly 5-CH_3_-tetrahydrofolate, which is metabolized in the liver. Some of the folic acid ingested might enter the blood circulation directly, depending on the dose [36]. Natural foods, such as vegetables and fruits, contain polyglutamate forms of reduced folates, with tetrahydrofolate being the predominant form [37]. Such polyglutamate forms of reduced folates are hydrolyzed to monoglutamate forms via the action of folylpoly-γ-glutamate carboxypeptidase, and are then absorbed in the intestine.

Proton-coupled folate transporter and reduced folate carrier are reportedly responsible for the mammalian intestinal absorption of folates [36,37]. *C. elegans* has orthologous genes encoding reduced folate carrier (*folt-1*) and folylpoly-γ-glutamate carboxypeptidase (*gcp-2.1*), which are involved in intestinal folate absorption [38,39]. The worm reduced folate carrier (FOLT-1) showed a very low affinity for folic acid because it is specific for reduced folates, similar to the mammalian carrier [39]. Folic acid is reportedly absorbed in the human intestine by the proton-coupled folate transporter [37]. Although its orthologous genes occur in *C. elegans,* the physiological functions of the gene products of these genes have been hardly characterized.

The 5-CH_3_-tetrahydrofolate that is transported into tissues must be metabolized to polyglutamate forms, which are retainable in cells via the action of folylpolyglutamate synthase. However, 5-CH_3_-tetrahydrofolate is a poor substrate for folylpolyglutamate synthase. Thus, 5-CH_3_-tetrahydrofolate has to be converted to tetrahydrofolate via the action of cobalamin-dependent methionine synthase [36]. In turn, the folic acid that is directly taken up by cells has to be reduced to tetrahydrofolate via the action of dihydrofolate reductase, because tetrahydrofolate is the adequate substrate for folylpolyglutamate synthase [36]. These observations indicate that cobalamin-dependent methionine synthase is the key enzyme in folate metabolism. Moreover, 5,10-methylenetetrahydrofolate reductase, which is involved in the synthesis of 5-CH_3_-tetrahydrofolate—a methyl donor for homocysteine remethylation to methionine—, is another key enzyme because its deficiency is associated with homocystinuria. Remarkably, chronic supplementation with high-dose folic acid significantly reduced the expression levels of the cobalamin-dependent methionine synthase reductase and 5,10-methylenetetrahydrofolate reductase mRNAs in *C. elegans* (Fig. 5). Cobalamin-dependent methionine synthase essentially requires methionine synthase reductase for the reactivation of the inactivated enzyme formed during the enzymatic reaction [40]. As shown in Fig. 4, folic acid was accumulated significantly in high-dose folic acid-supplemented worms. Folic acid could act as a potent inhibitor of cobalamin-dependent methionine synthase, which is involved in folate metabolism (Fig. 6). It is reported that excessive ingestion of folic acid appears to form an excess pool of unmetabolized folic acid because of the saturation and (or) inhibition of dihydrofolate reductase in mammals [41,42]. These results suggest that excess unmetabolized folic acid strongly induces disorders of both the folate and methionine cycles (Fig. 1). Consequently, the disruption of the methionine cycle by supplementation with high-dose folic acid induced a significant decrease in SAM level in worms (Fig. 7). SAM reportedly acts as an activator of cystathionine β-synthase, which is involved in the trans-sulfuration pathway of homocysteine, and is an allosteric inhibitor of 5,10-methylenetetrahydrofolate reductase, which is involved in the remethylation pathway of homocysteine [43]. Such metabolic regulations would be disrupted by the reduced SAM to inhibit the two homocysteine metabolic pathways, leading to the significant accumulation of unmetabolized homocysteine as a potent pro-oxidant (Fig. 8). Homocysteine is known to readily undergo self-oxidization to produce ROS [44] and can activate NADPH oxidase to generate ROS [45] because homocysteine stimulates the phosphorylation of NADPH oxidase subunits using protein kinase Cβ to increase oxidase activity. In particular, the accumulation of H_2_O_2_ in high-dose folic acid-supplemented worms is remarkable (Fig. 8, Fig. 9). Many studies [[46], [47], [48]] have reported that antioxidant enzymes (superoxide dismutase and catalase) have been potently inhibited by H_2_O_2_. Similarly, disrupted cellular redox homeostasis by the accumulation of unmetabolized homocysteine has also been reported in vitamin B_12_-deficient worms that showed significant reduction in the activity of cobalamin-dependent methionine synthase [49]. Moreover, the results reported above were similar phenomena to those observed in mammals during folate deficiency [50,51], which reportedly induces severe oxidative stress, leading to DNA damage [52,53] and increased blood pressure and insulin resistance [54] in mammals.

For the reduction of the risk of neural tube defects, the consumption of 400 μg of dietary folate equivalent per day from supplements or fortified foods is recommended in all women capable of becoming pregnant [36]. However, chronic high-dose folic acid supplementation significantly decreased the total number of eggs laid in worms (Fig. 3). Moreover, the knockout of *folt*-1 in *C. elegans* induced folate deficiency, leading to the sterility of hermaphrodites because of defects in germ-line proliferation [38]. These results suggest that the chronic intake of a mega-dose of folic acid might induce infertility in mammals.

## 5 Conclusions

Using *C. elegans* as a model animal, we examined the effects of chronic supplementation with high-dose folic acid to evaluate whether such folic acid supplementation is beneficial or harmful. High-dose folic acid supplementation significantly decreased egg-laying capacity of worms and accumulated unmetabolized folic acid was detected in the treated worms. Highly accumulated unmetabolized folic acid had the ability to inhibit cobalamin-dependent methionine synthase, which is involved in the folate and methionine cycles. Consequently, disorders of these metabolic pathways induced the accumulation of unmetabolized homocysteine, leading to severe oxidative stress. The current results, which were obtained after chronic supplementation of high-dose folic acid, matched the phenomena observed in mammals during folate deficiency.

## Author contributions

K.K. and Y.M. performed most experiments. K.K., T.B., Y.Y., and F.W. designed the experiments, analyzed data, and interpreted the results. K.K. wrote the original draft. T.B., Y.Y., and F.W. reviewed and edited the manuscript. All authors commented on the manuscript and approved the final version.

## Declaration of competing interest

The authors declare that they have no known competing financial interests or personal relationships that could have appeared to influence the work reported in this paper.
