# Supplementary material for: High-dose folic acid supplementation results in significant accumulation of unmetabolized homocysteine, leading to severe oxidative stress in Caenorhabditis elegans
Source: Redox Biol. 2020 Sep 15;37:101724. doi: 10.1016/j.redox.2020.101724 (PMC7509461; doi:10.1016/j.redox.2020.101724)
Supplement: Multimedia component 1 [file mmc1.docx]

**Supplemental material**

**High-dose folic acid supplementation results in significant accumulation of unmetabolized homocysteine, leading to severe oxidative stress in *Caenorhabditis elegans***

Kyohei Koseki,^1^ Yukina Maekawa,^2^ Tomohiro Bito,^1,2^ Yukinori Yabuta,^1,2^ Fumio Watanabe^1,2^

^1^ The United Graduate School of Agricultural Sciences, Tottori University, 4-101 Koyama-Minami, Tottori city, Tottori 680-8553, Japan

^2^ Graduate school of Sustainability Science, Tottori University, 4-101 Koyama-Minami, Tottori city, Tottori 680-8553, Japan

As porcine kidney powder, chicken pancreas powder, and rat serum are commercially available, they were used as crude sources of the folate conjugase enzyme [*1*, *2*]. Because considerable amounts of folates exist in these crude preparations, the internal folates must be removed by charcoal treatment or dialysis before use. Moreover, the specific activity of folate conjugases from chicken pancreas, rat serum, and porcine kidney is considerably low (8.2, 0.05, and 0.03 nmol/min/mg protein, respectively) (our unpublished data). Thus, we prepared a recombinant human folate conjugase with a high specific activity.

**Materials and methods**

***Materials***

The Human Reference cDNA was purchased from Takara Bio (Shiga, Japan). Pteroyl hepta-γ-l-glutamic acid (PteGlu_7_), pteroyl penta-γ-l-glutamic acid (PteGlu_5_), pteroyl tri-γ-l-glutamic acid (PteGlu_3_), pteroyl di-γ-l-glutamic acid (PteGlu_2_), and pteroyl mono-γ-l-glutamic acid (PteGlu_1_) were purchased from Schircks Laboratories (Jona, Switzerland). Other chemicals were purchased from FUJIFILM Wako Pure Chemical Corp. (Osaka, Japan).

***Expression and purification of recombinant human folate conjugase***

The cDNA fragment encoding human conjugase (Fig. S1), excluding the predicted signal peptide, was amplified from the qPCR Human Reference cDNA (Takara Bio) by PCR using the following primer sets: human folate conjugase-F (5′-CGCGCGGCAGCCATATGAGACCCCACGGCGACACC-3′) and human folate conjugase-R (5′-GGTGGTGGTGCTCGAGTCAATCAAATATGTAACATTGC-3′). Before ligation into the pET28b expression vector (Novagen, San Diego, CA, USA), the cDNA fragments were isolated by gel electrophoresis using the InFusion^®^ HD Cloning Kit (Takara Bio). DNA sequencing was performed using an automatic DNA sequencer (ABI Prism 3100 Genetic Analyzer; Applied Biosystems, Foster City, CA, USA).

After the *E. coli* BL21 Star^™^ (DE3) pLysS strain (Life Technologies, Carlsbad, CA, USA) was transformed with the pET28b/Hs conjugase vector, the transformants were grown at 37°C in 50 mL of Luria–Bertani (LB) medium containing 50 µg/mL kanamycin and 34 µg/mL chloramphenicol for 24 h. The whole culture was transferred to 1 L of Overnight Express^™^ Instant TB Medium (Novagen) and grown for 24 h at 37°C. *E. coli* cells were collected by centrifugation at 6,000 × *g* for 10 min, and stored at −30°C until use.

Recombinant *E. coli* cells (10.0 g) were suspended in 40 mL of 50 mM potassium phosphate, pH 7.0, containing 300 mmol/L NaCl and 5 mmol/L imidazole, disrupted (10 kHz) using a sonicator (10 s strokes at 50 s intervals), and centrifuged at 15,000 × *g* for 15 min. The His6-tagged recombinant human folate conjugase was purified on a column packed with TALON metal-affinity resin (Clontech Laboratories, Palo, Alto, USA). The enzyme was eluted with 50 mmol/L potassium phosphate, pH 7.0, containing 300 mmol/L NaCl and 150 mmol/L imidazole. The recombinant human folate conjugase obtained was stored at −30°C.

***SDS–PAGE***

SDS***–***PAGE was performed on 12.5% (w/v) polyacrylamide slab gels according to the method of Laemmli [*3*]. Samples were treated by boiling for 3 min in a 5% (v/v) 2-mercaptoethanol solution containing 0.1% (w/v) SDS. Proteins in the gel were stained with the CBB Stain One reagent (Nakarai tesque, Kyoto, Japan). Densitometric analysis was performed with the Image J software (Image Processing and Analysis in Java).

***Assay of human folate conjugase***

To determine the activity of recombinant human folate conjugase, enzymatic reaction products were analyzed using HPLC. The reaction mixture contained human folate deconjugase (30 µL), 100 µmol/L PteGlu_7_ (50 µL), and 100 mmol/L sodium acetate buffer, pH 4.5 (420 µL). The enzyme reaction was started by the addition of PteGlu_7_ and proceeded for 0, 10, 20, 30, 60, 120, and 180 min at 37°C. The enzymatic reaction was terminated by heat treatment for 5 min. The reaction mixture was centrifuged at 15,000 × *g* for 10 min at 4°C and the supernatant was filtered with a membrane filter (Miex®-LH, Merck Milipore). An aliquot (10 µL) of the reaction mixture was analyzed with on a Shimadzu HPLC apparatus, which consisted of an SPD-20A UV detector, an LC-20AB pump, a CTO-20AC column oven, a DGU-20A degasser, and a Luna C18(2) (φ4.6 × 250 mm) column (Shimadzu GLC Ltd.). The mobile phases were 0.1 mol/L acetate buffer (pH 5.5) and acetonitrile (solvent A). PteGlu_7_ and PteGlu_1_ were eluted with a linear gradient of acetonitrile (0% to 7.5% solvent A in 0–32 min, 7.5% solvent A in 32–37 min, and 7.5% to 0% solvent A in 37–42 min) at a flow rate of 0.8 mL/min at 30°C, and determined by measuring absorbance at 290 nm. The peak area was calculated using a data processor (CDS-Lite; LA soft, Chiba, Japan). The specific activity of recombinant folate conjugase was calculated based on the amount of PteGul_1_ formed over 60 min.

***Protein assay***

Protein was assayed by the method of Bradford [*4*] using ovalbumin as a standard.

**Results and discussion**

***Expression and purification of recombinant human folate conjugase***

A comparison of the deduced amino acid sequence of folate conjugases from various animals is shown in Fig. S1. Human folate conjugase has 77.4%, 68.7%, and 58.8% amino acid identities with porcine, rat, and chicken enzymes, respectively. The N-terminal amino acid sequence of rat folate conjugase reportedly contains a signal peptide (the first 24 amino acids) [*5, 6*]. Thus, the human folate conjugase gene without the moiety encoding the signal peptide was cloned, isolated, and expressed in *E. coli*.

SDS–PAGE of the *E. coli* extract showed that the induced band corresponding to the recombinant human folate conjugase has an apparent molecular mass of 36 kDa, which is identical to that calculated from the deduced amino acid sequence of human folate conjugase (Fig. S2). The recombinant human folate conjugase represented approximately 13.4% of the total proteins of the *E. coli* extract. A large quantity (approximately 30.9 mg of protein of the purified enzyme per 1 L of *E. coli* culture) of the purified enzyme was readily obtained. The purified human folate conjugase showed a high specific activity (330 nmol/min/mg protein).

***Properties of human folate conjugase***

The optimum temperature of the recombinant human folate conjugase was 30°C. The enzyme, when incubated at various temperatures for 10 min at pH 7.0, was stable up to 30°C, and the enzymatic activity was completely abolished at 70°C. The optimum pH of the recombinant human folate conjugase was 4.5, which was identical to that of the human plasma folate conjugase [7].

When PteGlu_7_ was treated with the recombinant human folate deconjugase for the indicated time, several intermediate products, such as PteGlu_5_, PteGlu_3_, and PteGlu_2_, were formed as the levels of PteGlu_7_ decreased up to 30 min. PteGlu_7_ was completely converted to PteGlu_1_ as the end product after 180 min of reaction (Fig. S3). This result suggets that human folate conjugase has exopeptidase activity, similar to the porcine kidney folate conjugase, whereas the chicken pancreas and rat serum folate conjugases have endopeptidase activity [*5*].

**References**

[1] Patring JD., Jastrebova JA., Hjortmo SB., Andlid TA., and Jagerstad IM. (2005) Development of a simplified method for the determination of folates in baker’s yeast by HPLC with ultraviolet and fluorescence detection. J Agric Food Chem. 53: 2406-2411.

[2] [Devries JW](https://www.ncbi.nlm.nih.gov/pubmed/?term=DeVries%20JW%5BAuthor%5D&cauthor=true&cauthor_uid=15759720)., [Rader JI](https://www.ncbi.nlm.nih.gov/pubmed/?term=Rader%20JI%5BAuthor%5D&cauthor=true&cauthor_uid=15759720)., [Keagy PM](https://www.ncbi.nlm.nih.gov/pubmed/?term=Keagy%20PM%5BAuthor%5D&cauthor=true&cauthor_uid=15759720)., [Hudson CA](https://www.ncbi.nlm.nih.gov/pubmed/?term=Hudson%20CA%5BAuthor%5D&cauthor=true&cauthor_uid=15759720)., [Angyal G](https://www.ncbi.nlm.nih.gov/pubmed/?term=Angyal%20G%5BAuthor%5D&cauthor=true&cauthor_uid=15759720)., [Arcot J](https://www.ncbi.nlm.nih.gov/pubmed/?term=Arcot%20J%5BAuthor%5D&cauthor=true&cauthor_uid=15759720)., [Castelli M](https://www.ncbi.nlm.nih.gov/pubmed/?term=Castelli%20M%5BAuthor%5D&cauthor=true&cauthor_uid=15759720)., [Doreanu N](https://www.ncbi.nlm.nih.gov/pubmed/?term=Doreanu%20N%5BAuthor%5D&cauthor=true&cauthor_uid=15759720)., [Hudson C](https://www.ncbi.nlm.nih.gov/pubmed/?term=Hudson%20C%5BAuthor%5D&cauthor=true&cauthor_uid=15759720)., [Lawrence P](https://www.ncbi.nlm.nih.gov/pubmed/?term=Lawrence%20P%5BAuthor%5D&cauthor=true&cauthor_uid=15759720)., [Martin J](https://www.ncbi.nlm.nih.gov/pubmed/?term=Martin%20J%5BAuthor%5D&cauthor=true&cauthor_uid=15759720)., [Peace R](https://www.ncbi.nlm.nih.gov/pubmed/?term=Peace%20R%5BAuthor%5D&cauthor=true&cauthor_uid=15759720)., [Rosner L](https://www.ncbi.nlm.nih.gov/pubmed/?term=Rosner%20L%5BAuthor%5D&cauthor=true&cauthor_uid=15759720)., [Strandler HS](https://www.ncbi.nlm.nih.gov/pubmed/?term=Strandler%20HS%5BAuthor%5D&cauthor=true&cauthor_uid=15759720)., [Szpylka J](https://www.ncbi.nlm.nih.gov/pubmed/?term=Szpylka%20J%5BAuthor%5D&cauthor=true&cauthor_uid=15759720)., [van den Berg H](https://www.ncbi.nlm.nih.gov/pubmed/?term=van%20den%20Berg%20H%5BAuthor%5D&cauthor=true&cauthor_uid=15759720)., [Wo C](https://www.ncbi.nlm.nih.gov/pubmed/?term=Wo%20C%5BAuthor%5D&cauthor=true&cauthor_uid=15759720)., and [Wurz C](https://www.ncbi.nlm.nih.gov/pubmed/?term=Wurz%20C%5BAuthor%5D&cauthor=true&cauthor_uid=15759720). (2005) Microbiological assay-trienzyme procedure for total folates in cereals and cereal foods: collaborative study. J AOAC Int. 88: 5-15.

[3] Laemmli UK*.* (1970) Cleavage of structural proteins during the assembly of the head of bacteriophage T4*.* Nature 277: 680*–*685*.*

[4] Bradford MM. (1976) A rapid and sensitive method for the quantitation of microgram quantities of protein utilizing the principle of protein-dye binding. *Anal. Biochem.* 72, 248-254.

[5] Yao R., Schneider E., Ryan TJ., and Galivan J. (1996) Human γ-glutamyl hydrolase: Cloning and characterization of the enzyme expressed in vitro. Proc. Natl. Acad. Sci. 93, 10134-10138.

[6] Yao R., Nimec Z., Ryan T., and Galivan J. (1996) Identification, cloning, and sequencing of a cDNA coding for rat γ-glutamyl hydrolase. *J. Biol. Chem*. 271, 8525-8528.

[7] Goli DM, and Vanderslice JT. (1992) Investigation of the conjugase treatment procedure in the microbiological assay of folate. Food Chem. 43, 57-64.

**

**

**Fig. S1. Deduced amino acid sequences of human, porcine, rat and chicken conjugases.**

Gaps were introduced to optimize the alignment. Amino acid residues found at the same position compared with human folate conjugase are shown as shaded characters. The arrowhead indicates the processing site of human folate conjugase. The amino acids indicated in bold in human folate conjugase indicate the signal peptide site [*5, 6*].


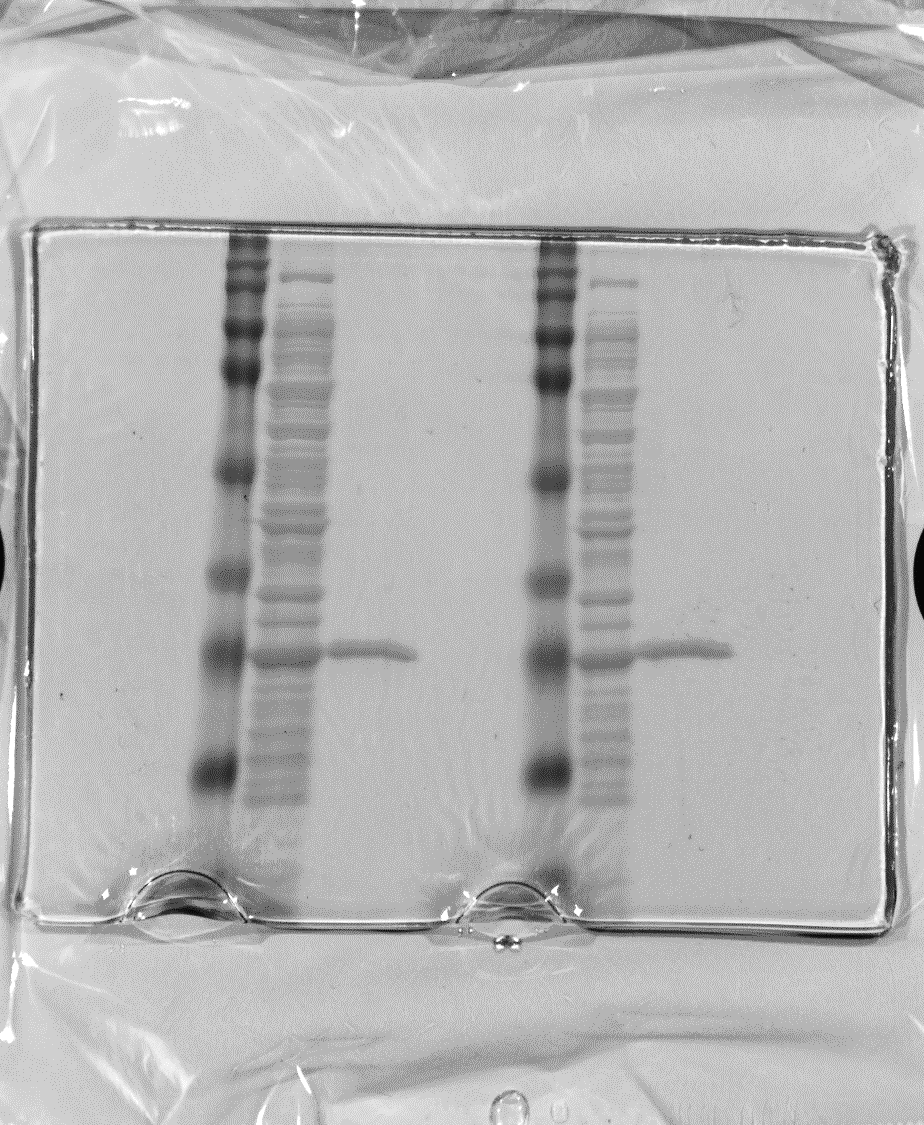


(kDa)

76

52

38

31

24

17

102

150

225

12

1

2

3

**Fig. S2.** **SDS–PAGE of purified recombinant human folate conjugase**

Lane 1, protein standards; lane 2, crude extracts of recombinant *E. coli* cells (40 µg); lane 3, purified recombinant human folate conjugase protein (4 µg).

Peak area

Reaction time (min)

**Fig. S3. Effect of reaction times on the products of recombinant human folate conjugase.**

● (PteGlu_7_), ◇ (PteGlu_5_), ▵ (PteGlu_3_), ▿ (PteGlu_2_), and ■ (PteGlu_1_).
